# Consensus statement on smoking cessation in patients with pain

**DOI:** 10.1007/s00540-022-03097-w

**Published:** 2022-09-07

**Authors:** Hiroki Iida, Shigeki Yamaguchi, Toru Goyagi, Yoko Sugiyama, Chie Taniguchi, Takako Matsubara, Naoto Yamada, Hiroshi Yonekura, Mami Iida

**Affiliations:** 1 Working Group on the Role of Smoking Cessation in Pain Relief, The Japan Society of Pain Clinicians (JSPC), Tokyo, Japan; 2 Department of Anesthesiology and Pain Medicine, Gifu University Graduate School of Medicine, Gifu, Japan; 3 Department of Anesthesiology, Dokkyo Medical University School of Medicine, Tochigi, Japan; 4 Department of Anesthesiology, Akita University Hospital, Akita, Japan; 5 Department of Woman Doctor Active Support in Perioperative Medicine, Gifu University Graduate School of Medicine, Gifu, Japan; 6 College of Nursing, Aichi Medical University, Nagakute, Japan; 7 Department of Physical Therapy, Faculty of Rehabilitation, Kobe Gakuin University, Kobe , Japan; 8 Department of Anesthesiology, Iwate Medical University Hospital, Iwate, Japan; 9 Department of Anesthesiology and Pain Medicine, Fujita Health University Bantane Hospital, Nagoya, Japan; 10 Department of Internal Medicine, Gifu Prefectural General Medical Center, Gifu, Japan; 11 Anesthesiology and Pain Relief Center, Central Japan International Medical Center, 1-1 Kenkonomachi, Minokamo, Gifu 505-8510 Japan

**Keywords:** Acute pain, Chronic pain, Smoking cessation, Withdrawal, Nicotine

## Abstract

Smoking is closely associated with the development of various cancers and tobacco-related illnesses such as cardiovascular and respiratory disorders. However, data are scarce on the relationship between smoking and both acute and chronic pain. In addition to nicotine, tobacco smoke contains more than 4000 different compounds. Although nicotine is not the sole cause of smoking-induced diseases, it plays a critical role in pain-related pathophysiology. Despite the acute analgesic effects of nicotine, long-term exposure leads to tolerance and increased pain sensitivity due to nicotinic acetylcholine receptor desensitization and neuronal plastic changes. The purpose of smoking cessation interventions in smoking patients with pain is primarily not only to reduce their pain and associated limitations in activities of daily living, but also to improve the outcomes of underlying pain-causing conditions and reduce the risks of tobacco-related disorders. This statement aims to summarize the available evidence on the impact of smoking on pain and to inform medical professionals of the significance of smoking cessation in patients with pain.

## Basic principles and overview

### Purpose of this document

Smoking is closely associated with the development of various diseases, including the following: lung, laryngeal, and other cancers; myocardial infarction, stroke, and other cardiovascular diseases; and respiratory disorders such as chronic obstructive pulmonary disease and asthma. However, data are scarce on the relationship between smoking and acute and chronic pain. Smoking patients are often refractory to pain control. Until recently, there was a lack of consensus regarding smoking treatment options that optimize pain relief.

This statement aims to summarize available evidence on the impact of smoking on pain, inform clinicians and other health care professionals of the significance of smoking cessation in patients with pain, and describe the role of smoking cessation in multidisciplinary pain management.

### Target audience

This document is intended for healthcare professionals who manage pain in smoking patients and those who provide smoking cessation therapies for patients with pain. Smoking patients who visit general physicians, surgeons, and other specialists often complain of pain. This document will help physicians, nurses, pharmacists, physical therapists, and other healthcare workers deal with smoking patients in their daily work.

## Levels of evidence and grades of recommendations

### Search strategy

In July 2020, we conducted a systematic search of the literature on smoking cessation and pain relief using MEDLINE (PubMed). Search terms included “smoking,” “tobacco,” “smoking prevention,” “smoking cessation,” “smoking cessation aid,” “nicotine,” “tobacco use disorder,” “analgesia,” “analgesic,” and “pain.” The literature search covered the period from January 2000 to June 2020. The specific search formulas are shown in the Appendix. In addition, references cited in the articles identified by the MEDLINE literature search were examined, where appropriate, when preparing answers to each clinical question. Our search was limited to studies published in English or Japanese. Published clinical guidelines and systematic reviews were assessed for quality, recency, and relevance before adopting them.

### Quality of evidence and strengths of recommendations

The quality of evidence and strength of a recommendation were assessed based on the “Minds Manual for Guideline Development 2017” [1] and “GRADE System for Clinical Practice Guideline, Third Edition” [2].

The overall quality of evidence on outcomes was categorized as follows:

- **A (high):** We are very confident that the true effect lies close to that of the estimate of the effect.
- **B (moderate):** We are moderately confident in the effect estimate.
- **C (low):** Our confidence in the effect estimate is limited.
- **D (very low):** We have very little confidence in the effect estimate.

The strength of a recommendation was assessed based on four factors: overall quality of evidence, balance between desirable and undesirable effects, values and preferences, and use of costs and resources. The strength of a recommendation was categorized and presented as follows:

- **1: Strong**
- **2: Weak**

Because of the nature of research, most studies on smoking cessation related to pain relief were cohort based, and there was a limited number of randomized controlled studies and systematic reviews. When the findings of our systematic literature search could not be formulated, we made no recommendation and did not indicate their strength or evidence quality.

Each recommendation is accompanied by its strength (1 or 2) and evidence quality (A, B, C, or D).

The study designs of individual references were classified into the categories below. As supplementary information, the category of each study is shown in brackets following its citation in the Reference Section.

- **Ia:** Systematic review/meta-analysis
- **Ib:** Randomized controlled trial
- **IIa:** Non-randomized controlled trial or large-scale cohort study (*n* ≥ 500)
- **IIb:** Analytic epidemiologic study: small-scale cohort, case-control, or cross-sectional
- **III:** Descriptive study: case report or case series report
- **IV:** Laboratory experiments and others

### Criteria for recommendations and consensus building

A draft version of this document was distributed to each Working Group member for review and comments. Working Group members were asked to confirm the text. After feedback comments were received, a consensus building meeting was held with all members in attendance. Consensus on each proposed recommendation was reached when at least 80% of members agreed. Discussions continued until the consensus criteria were met.

### Public comments from related academic societies

The Working Group solicited public comments on the draft version of this document from members of the Japan Society of Pain Clinicians (JSPC) and related academic societies. The Working Group discussed the comments received and decided whether to accept or reject them.

## Basic physiology of pain and tobacco smoking

### Objectives of smoking cessation in patients with pain

The primary objective of smoking cessation interventions in smoking patients is to alleviate their pain and associated limitations in activities of daily living (ADLs). Other objectives include improving the outcomes of the underlying pain-causing conditions and reducing the risks of tobacco-related disorders. Thus, smoking cessation interventions contribute to the goal of helping people lead healthy and fulfilling lives and improving their life expectancy.

### Pain and smoking

**Fig. 1:**
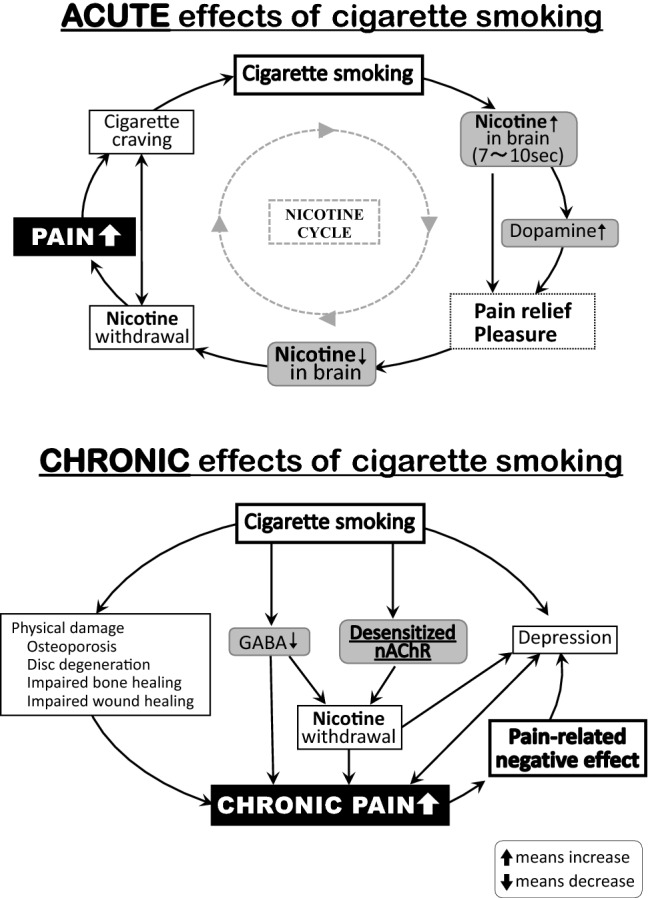
Acute and Chronic Effects of Cigarette Smoking. Top: Acute effects of cigarette smoking. Smoking rapidly increases brain concentrations of nicotine (7–10 s). Nicotine binds to nAChRs in the midbrain (ventral tegmental area), and this binding activates dopamine release at nerve endings in the nucleus accumbens. This dopamine release causes feelings of pleasure, euphoria, and pain relief [10]. However, brain nicotine levels rapidly decrease, with an elimination half-life of approximately 30 min. This rapid decrease lowers the cerebral levels of dopamine and other neurotransmitters, leading to nicotine withdrawal symptoms, greater pain intensities, and stronger craving for smoking [11]. One cycle of smoking, reward, withdrawal, and craving often leads to another. Bottom: Chronic effects of cigarette smoking. Long-term smoking may cause osteoporosis, disc degeneration, delayed bone and wound healing, and other physical damage [11], and also decrease GABA secretion, desensitize nAChRs, and enhance depressive tendencies [11–13]. Interactions between these changes can aggravate chronic pain. Moreover, nicotine withdrawal symptoms appear between cigarettes, and exacerbate chronic pain [14, 15]. The nicotine-induced pain exacerbation may further intensify depressive symptoms and chronic pain, thereby contributing to the vicious cycle of smoking [12, 14, 15]. *GABA* gamma-aminobutyric acid, *nAChR* nicotinic acetylcholine receptor

Tobacco smoke contains nicotine, carbon monoxide, and more than 4000 other compounds. Although nicotine is not the sole cause of smoking-induced diseases, it plays a critical role in pain-related pathophysiology. Its effects on pain perception are complex. Experimental and human models show that nicotine has acute analgesic effects that are mediated by the activation of nicotinic acetylcholine receptors (nAChRs) in the central and peripheral nervous systems [3–6]. These receptors control the release of noradrenaline, endogenous opioids, dopamine, and other neurotransmitters that activate the descending pain modulatory pathway or suppress the nociceptive input to the spinal dorsal horn [7, 8]. Long-term exposure to nicotine induces tolerance due to nAChR desensitization and neuronal plastic changes, which together increase the amounts of nicotine to achieve desired effects. Since nicotine is rapidly eliminated from the brain, its effects on pain reduction become more complicated under chronic exposure conditions, where the impacts of nicotine withdrawal symptoms and nAChR desensitization outweigh the analgesic effects of absorbed nicotine, thereby resulting in elevated pain sensitivity [9] (Fig. 1).

In rat models, pain thresholds change with the duration of nicotine exposure. They increase after 1 to 3 weeks of exposure, but decrease after ≥ 6 weeks of exposure [12]. In a rat model of neuropathic pain induced by sciatic nerve ligation, chronic nicotine exposure caused greater mechanical hyperalgesia than control treatment [16]. Moreover, rats chronically exposed to nicotine exhibited decreased pain thresholds and hyperalgesia following nicotine withdrawal [17, 18], suggesting the involvement of α7-type nAChRs (α7-nAChRs) [19]. Increased pain perception associated with nicotine withdrawal may involve changes in peripheral and central nervous system reactions (sensitization), endogenous opioid release, and hypothalamic–pituitary–adrenal (HPA) axis regulation [11].

Tobacco smoking has limited acute short-term analgesic effects. In special circumstances, nicotine may be administered as an analgesic in nonsmokers. However, analgesic effects of tobacco smoking are limited and short-term. Generally, individuals chronically exposed to tobacco smoke are strongly affected by the interplay between nicotine-induced temporary analgesic effects and increased pain sensitivity due to nicotine withdrawal [10, 14]. Complex smoking and pain relationships may develop as a result of interactions involving the following factors: (i) alteration in pain processing due to chronic nicotine exposure; (ii) interaction with opioids and other analgesics; (iii) structural damage to skeletal and other systems, resulting for instance in osteoporosis, spinal degenerative disease, delayed bone, and wound healing; (iv) development or worsening of depression and other psychiatric symptoms, and (v) poor socioeconomic conditions [11]. As a result of these and other factors, pain sensitivity increases after long-term nicotine exposure.

In Sect. 4, we discuss various tobacco-related problems in detail.

## Clinical questions

### Effects of smoking on pain (pathology)

#### Does smoking affect pain sensation?

**< Summary Statements >**

- Smoking and nicotine administration provide acute analgesic effects.
- Pain threshold and tolerance levels differ between smokers and nonsmokers.
- Nicotine withdrawal increases pain in smokers.

**< Commentary >**

Receptors of nicotine, the key component of tobacco smoke, are widely distributed in the central and peripheral nervous systems, and are involved in the physiology of arousal, sleep, anxiety, cognition, and pain. Nicotine activation of nAChRs gives rise to acute analgesic effects in animal models and humans [11]. In humans undergoing experimental pain induction, nicotine or tobacco administration conferred acute analgesic effects of small-to-moderate magnitude [10]. A meta-analysis of studies on intranasal or transdermal nicotine for the treatment of postoperative pain showed a reduction in postoperative pain at 24 h compared with placebo, but the effects were relatively small [20].

However, chronic exposure to nicotine, as observed in long-time smokers, is associated with complex changes in pain threshold and tolerance. Girdler et al. reported that female smokers had a greater ischemic pain threshold than female nonsmokers, and male smokers had a greater cold pressor pain threshold than male nonsmokers [21]. Smokers showed evidence for blunted HPA-axis function during stress, as well as compromised endogenous pain regulatory mechanisms. Duan et al. found that whereas smokers had a higher preoperative pressure pain threshold than nonsmokers, the incidence of inadequate postoperative analgesia was significantly greater in smokers [22]. Female smokers had significantly lower cold pressor pain tolerance than both female nonsmokers and smoking and nonsmoking men [23].

Nicotine withdrawal enhances pain perception. Smokers experienced greater pain during the first 12–24 h of smoking abstinence [14, 15]. The levels of nicotine dependence were positively associated with greater pain intensity [24, 25]. Smokers abstinent for < 1 month had lower thresholds for electrically evoked pain than nonsmokers. These findings indicate that nicotine withdrawal lowers pain thresholds and causes hyperalgesia in long-term smokers who have been chronically exposed to and dependent on nicotine. Since it is not literally possible for smokers to keep smoking throughout the day to prevent withdrawal symptoms, nicotine withdrawal that occurs during abstinence unavoidably elevates the risk of hyperalgesia.

#### Is smoking associated with pain?

**< Summary Statements >**

- Smokers have a higher prevalence of chronic pain and greater pain intensity than nonsmokers.
- Smoking exacerbates nociceptive, neuropathic, and psychosocial pain.
- Acute pain is more likely to become chronic in smokers.
- Smokers tend to experience worsening of pain-related pathologies (e.g., mood disorders, depression, and sleep disturbance), ADLs, and social activities.
- Smoking increases the risks of osteoporosis, disc degeneration, fractures, and lumbago.

**< Commentary >**

Estimated prevalence rates of current smoking in patients with pain ranged from 42 to 68%, which may be up to twice that observed in the general population (including patients with non-chronic pain and healthy individuals) [26]. The prevalence rates of chronic pain in former smokers (35–49%) were higher than in nonsmokers, irrespective of age and sex [26]. In addition, chronic pain was more severe in smokers than in nonsmokers and former smokers, as measured by pain frequency, intensity, duration, and total number of pain sites [27].

Regarding the prevalence of nociceptive pain, a large-scale cohort study reported that smoking was associated with a 20% increase in the risk of musculoskeletal pain (odds ratio [OR]: 1.23) [28]. Moreover, the prevalence of chronic pain symptoms such as spinal pain, fibromyalgia, and head pain was also higher in women who smoked daily than in nonsmoking women (OR: 2.04) [29]. The odds of developing rheumatoid arthritis were approximately twice as high in current smokers than in nonsmokers, and four times higher in rheumatoid factor–positive current smokers [30]. Regarding pain severity, studies on spinal and temporomandibular pain reported significantly higher visual analogue scale (VAS) pain scores in smokers than in nonsmokers, and heavy smokers had a significantly greater risk of rheumatoid factor–positive rheumatoid arthritis than nonsmokers (OR: 3.06) [31–33]. Previous studies generally indicated that smoking was associated with higher levels of inflammatory nociceptive pain, irrespective of whether the pain sensitivity was measured using the VAS, a numerical rating scale, or other methods.

The odds of experiencing neuropathic pain were also higher in smokers than in nonsmokers (e.g., postherpetic neuralgia OR, 1.62–2.08; sciatica OR, 2.01), and current or former smoking status was significantly associated with intense or very intense pain at the onset of herpes zoster (OR: 2.00) [34, 35].

Although our literature search did not identify studies that investigated smoking-related psychosocial pain using the VAS, a numerical rating scale, or similar scales, it is reasonable to believe that smoking increases the prevalence and intensity of psychosocial pain because smoking exacerbates psychological problems, as described below. Social environmental factors, such as family and personal history, occupational functioning, and social support, play an important role in the cause, course, and outcomes of pain and smoking [26]. Lower socioeconomic status and educational levels were also independently associated with greater pain and increased cigarette consumption [26].

Moreover, current and previous smoking was an independent predictive factor of the prevalence and intensity of chronic pain (e.g., neuropathic, facial, temporomandibular, and abdominal) [27].

Research using functional magnetic resonance imaging showed that smoking altered brain functional connectivity of the basal ganglia, resulting in pain chronification [36]. A longitudinal study showed that current smoking status at baseline was an independent predictor of chronic pain persisting for ≥ 10 years (OR: 1.43) [37]. Moreover, a significant dose–response relationship was observed between smoking and chronic pain intensity [38].

Compared with their nonsmoking counterparts, smoking patients with pain were at greater risks of mental health problems (anxiety, depression, catastrophic thinking), sleep problems (daytime fatigue, somnolence, poor sleep quality), abuse of alcohol and other substances, and suicidal ideation [39, 40]. Smoking may also delay the recovery of physical abilities such as daily walking and fine upper-limb motor skills, which may interfere with post-injury employment and social life [41–43].

Furthermore, smoking was associated with changes to the bone and surrounding structures, increasing the lifetime risks of developing osteoporosis, disc degeneration, fractures, and low back pain [44, 45].

#### Is passive smoking associated with pain?

**< Summary Statements >**

- Passive smoking increases postoperative pain intensity.
- Passive smoking increases the prevalence of chronic pain.
- Passive smoking aggravates disc degeneration and low back pain.
- Passive smoking in pregnant women is associated with the development of hyperalgesia in their infants.

**< Commentary >**

Among patients who underwent open lobectomy surgery, those exposed to secondhand smoke had significantly lower postoperative arterial oxygen tension than nonsmoking controls, and required larger amounts of morphine consumption [46]. In patients undergoing abdominal hysterectomy, passive smoking was associated with greater postoperative VAS pain intensity scores and larger doses of fentanyl-based patient-controlled analgesia compared with nonsmoking [47].

In a retrospective population-based study in Denmark, both active and passive smoking were associated with higher prevalence rates of back pain and other types of chronic pain [38]. In a prospective study of children undergoing venous catheterization, passive smoking increased intraoperative pain perception [48]. A cohort study in Hong Kong showed that passive smoking, particularly in public outdoor places, was associated with lower physical and mental health scores, including body pain [49].

Studies in humans [50] and animals [51, 52] showed that passive smoking elevated the risk of disc degeneration. In a prospective cross-sectional study of female high school students, passive smoking was associated with the incidence of low back pain during the previous month [53]. Of note, however, a retrospective epidemiological study that examined the relationship between nonspecific low back pain and environmental and life-style factors in children aged 7.5 to 14 years did not identify passive smoking as a significant etiological factor [54].

In a cohort study of passive smoking in Singapore, the prevalence of teething pain in infants was significantly associated with their mothers’ exposure to passive smoking during early pregnancy [55]. Another study reported that intrauterine exposure to tobacco smoke was associated with the pain perception levels of newborns, as determined during routine vaccination injections given at 48 h after birth [56]. These findings suggest that passive smoking in pregnant women affects the pain perception of their infants.

#### Do novel tobacco products influence pain in patients?

**< Summary Statements >**

- Heated tobacco products (HTPs) should be treated in the same way as conventional cigarettes because they contain nicotine, which increases pain sensitivity.

**< Commentary >**

HTPs release tobacco components by directly heating tobacco leaf or passing vaporized glycerol or other solvents over tobacco leaf. From the perspectives of tobacco and nicotine regulations in Japan, they are completely different from nicotine-containing electronic cigarettes (e-cigarettes), which are gaining increasing popularity overseas. Nicotine is classified as a pharmaceutical agent in Japan under the Pharmaceuticals and Medical Devices Act (Act on Securing Quality, Efficacy and Safety of Pharmaceuticals, Medical Devices, Regenerative and Cellular Therapy Products, Gene Therapy Products, and Cosmetics). Currently, nicotine-containing e-cigarettes are not commercially available in Japan.

HTPs release aerosols containing nicotine at concentrations that differ from one product to another [57, 58]. Controlled confinement studies showed that plasma nicotine levels on the fifth day of confinement were comparable between HTP users and cigarette smokers [59].

Several reports have been published regarding the relationship between e-cigarette use and pain. Compared to smokers who reported no pain in the past 2 weeks, those with significant pain were 3 times more likely to use e-cigarettes and a greater number of nicotine products, and were approximately 4 times more likely to have tried e-cigarettes and 7 times more likely to have tried cigars [60]. A study of e-cigarette users showed that pain experience was related to the severity of their e-cigarette dependence and to their perceived barriers to quitting e-cigarettes [61].

In a population survey conducted in the United States, a substantial increase in e-cigarette use was associated with a significant increase in smoking cessation rate [62]. Some may argue that nicotine-based e-cigarettes provide a type of nicotine replacement therapy, and thereby contribute to smoking cessation because they generate nicotine-containing vapors that are free from harmful substances resulting from combustion or heating of tobacco leaf. However, the US Centers for Disease Control and Prevention has warned that e-cigarettes can cause serious acute lung injuries, termed “e-cigarette or vaping product use associated lung injury” (EVALI), and suggested that these injuries are attributable to tetrahydrocannabinol (a psychoactive ingredient in marijuana regulated in Japan), cannabidiol (CBD), and vitamin E acetate present in e-liquids. In Japan, CBD-containing products are available for use in e-cigarettes and HTPs, warranting close attention. E-cigarette products that are marketed in Japan do not contain nicotine, and thereby appear to have low efficacy for smoking cessation [63].

#### Does smoking influence postoperative pain?

**< Summary Statements >**

**Recommendation:** We strongly recommend smoking cessation because smoking is a significant risk factor for poor control of acute postoperative pain. **[1C].**

- Nicotine administration has only negligible effects on alleviating acute postoperative pain.
- Smokers show higher acute pain intensity scores postoperatively than nonsmokers, and require greater opioid analgesic doses. These trends are more pronounced with higher levels of nicotine dependence.
- Patients show lower postoperative acute pain scores when starting smoking abstinence ≥ 3 weeks before surgery than when starting it immediately before surgery.
- Smokers are at greater risk than nonsmokers for chronic postoperative pain and long-term use of opioids and other analgesics.

**< Commentary >**

Based on evidence of generally low quality, a Cochrane review of acute postoperative pain reported that nicotine formulated as a nasal spray or transdermal patch could reduce pain scores at 24 h postoperatively compared with placebo, but the effects were relatively small (0.88 points on a 0–10 pain scale) [20]. Nicotine has limited effects on acute postoperative pain relief. In a prospective study of patients undergoing elective surgery under general anesthesia, male current smokers had worse VAS pain intensity scores postoperatively and required larger opioid doses than male nonsmokers and former smokers [64]. In a meta-analysis that investigated the causes of poor acute postoperative pain control based on 33 clinical studies, preoperative smoking was an independent predictor [65]. The Table 1 summarizes other studies that demonstrated the impact of smoking on acute postoperative pain intensity or opioid analgesic requirements in patients undergoing a variety of surgical procedures.

**Table 1:** Effects of smoking on acute postoperative pain intensity

Procedure		Increased acute pain score	Increased opioid analgesic dose
Locomotor	Rotator cuff repair	Cuff et al. [66]	
	Hip replacement	Buzin et al. [67]	
	Knee replacement		Wojahn et al. [68]
	Foot and ankle surgery		Mulligan et al. [69]
	Orthopedic surgery in general		Steinmiller et al. [70]
Head and neck	Thyroid and parathyroid surgery		Chen et al. [71]
	Otolaryngological surgery		Dang et al. [72]
Chest	Thoracoscopic surgery	Sun et al. [73]	
	Thoracic surgery	Yu et al. [74]	Yu et al. [74]
Abdomen	Distal gastrectomy		Kim et al. [75]
	Hepatectomy	Shen et al. [76]	Shen et al. [76]
	Gynecologic pelvic surgery		Woodside [77]

In a study by Yu et al. of patients undergoing thoracic surgery, postoperative pain severity and patient-controlled intravenous sufentanil requirement increased with increasing nicotine dependence [74]. In patients with high nicotine dependence who had radical resection of lung cancer, preoperative smoking cessation within 3 weeks of surgery resulted in worse postoperative pain outcomes and greater opioid requirement than earlier smoking cessation [78]. Even those who stopped smoking ≥ 3 weeks before surgery had worse postoperative pain intensity scores and greater opioid requirements than nonsmokers. These findings underscore the need for a sufficient duration of smoking abstinence before surgery to minimize the adverse effects of smoking.

Smokers are at greater risk of not only acute postoperative pain but also of chronic pain ≥ 3 months after surgery. Smoking was associated with the development of chronic pain after breast surgery [79], and it increased pain intensity scores at 15 months postoperatively [80]. Smoking was also associated with chronic postoperative pain in patients treated with hysterectomy [81], total shoulder arthroplasty [82], and nonunion surgery [83]. Moreover, smoking was associated with opioid analgesic use for ≥ 3 months postoperatively in various orthopedic surgeries, including hip replacement [84], shoulder arthroplasty [82, 85], and spine surgeries [86, 87], as well as in cardiothoracic [88], urologic [89], and kidney donation surgeries [90]. Smoking is a significant predictor of opioid abuse [91, 92].

The "Practical Guide for Perioperative Smoking Cessation” published by the Japanese Society of Anesthesiologists states that smoking is associated with various postoperative respiratory and other complications such as delayed wound healing and surgical site infection, and strongly recommends preoperative smoking cessation to reduce perioperative risks [93] (evidence level: B).

The recommendation was based on the result of systematic review and meta-analysis [65]. The overall quality of evidence is low because of a risk of bias and the indirectness of the results. The desirable effects of smoking cessation clearly outweigh the undesirable effects. Therefore, we strongly recommend smoking cessation because smoking is a significant risk factor for poor control of acute postoperative pain. [1C].

#### Does smoking affect cancer-related pain?

**< Summary Statements > **

- Cancer-related pain increases when patients continue to smoke after being diagnosed with cancer.
- Cancer patients who smoke have a greater need for opioid analgesics after surgery than nonsmoking patients, and are more prone to pain chronification.
- Smoking increases the prevalence of chemotherapy-induced peripheral neuropathies.
- Smoking increases the incidence of painful side effects of cancer radiation therapy.
- Among patients with advanced cancer, smokers show higher pain intensity scores and require larger doses of opioid analgesics than nonsmokers.

**< Commentary >**

Smoking increases preoperative and postoperative pain in cancer patients undergoing surgery. It also increases pain in patients receiving chemotherapy and radiation therapy. Moreover, it increases pain in patients with advanced cancer. Some studies have reported that smoking cessation lowers pain intensity.

Multiple observational studies showed that patients who continued to smoke after cancer diagnosis had more severe pain than nonsmokers and former smokers [94, 95]. In addition, smoking that continued after cancer diagnosis increased the likelihood of pain treatment [96]. One cross-sectional study and one small-scale cohort study indicated that smoking cessation after cancer diagnosis reduced pain [97]. Regarding the relationship between cancer pain and pre- and postoperative smoking, nonsmokers had less opioid consumption in the immediate postoperative period [78, 98], while smokers experienced greater chest and wound pain as well as a persistent decrease in physical and social functioning postoperatively [99]. Since nonsmokers had a higher postoperative quality of life (QOL) [99], patients undergoing surgery should be smoke-free to minimize the postoperative risks of pain chronification and functional impairments.

In patients undergoing radiation therapy or chemotherapy, smoking is associated with greater incidences of side effects and elevated levels of pain. Specifically, smoking is an identified risk factor for chemotherapy-induced peripheral neuropathies [100], oral pain due to mucositis and other adverse events in oral cancer patients undergoing radiation therapy [101], and severe skin toxicities in breast radiation therapy [102, 103].

Among patients with advanced cancer, smokers reported significantly higher degrees of pain, fatigue, anorexia, depression, anxiety, and insomnia than nonsmokers, and required larger doses of opioid analgesics [104, 105]. Smokers with advanced cancer had a lower QOL due to stronger pain intensities [106].

### Effects of smoking on pain (pain treatment)

#### Can pain be effectively managed in smoking patients?

**< Summary Statements > **

- Smokers are more likely to exhibit poor medication adherence and overdose than nonsmokers.
- Pain treatment outcomes are poorer in smokers than in nonsmokers.
- Smokers show poorer outcomes of multidisciplinary pain management and lower rates of return to work.
- Smoking interferes with effective pain control because pain worsens even after short periods of smoking deprivation.

**< Commentary >**

Smokers require greater opioid doses for pain relief than nonsmokers, but despite their higher doses, they show greater pain intensity scores. This is the case for both acute and chronic pain, including acute postoperative pain [78] and chronic low back pain [107]. In a study of hydrocodone use in patients with chronic pain, smokers required larger doses but had less successful pain control and lower serum hydrocodone levels than nonsmokers [107]. In studies of duloxetine in smoking and nonsmoking psychiatric patients (not directly related to pain relief), serum levels of the drug were lower in smokers than in nonsmokers, suggesting that tobacco smoke components induce drug-metabolizing enzymes (e.g., cytochrome P450 1A2) [108, 109]. In a study of chronic pain medication use, smokers showed poor pain medication adherence, and overuse was associated with current smoking [110]. Smokers had a greater likelihood of long-term opioid use after major surgeries [86, 111].

Smokers showed poorer outcomes of non-pharmacologic pain interventions such as nerve block [112] and spinal cord stimulation [113]. They also showed less satisfactory outcomes than nonsmokers in spinal surgery [114, 115] and arthroplasty [116] in terms of pain relief, patient satisfaction, and QOL. Smokers were at greater risk of undergoing revision total knee arthroplasty [117]. Moreover, multidisciplinary pain treatment programs yielded lower completion rates [118] and lower rates of return to work [119] in smokers than in nonsmokers. Smokers generally have more difficulty controlling pain than nonsmokers.

Daily tobacco smokers often experience greater pain with short periods of smoking deprivation [14]. Smoking is considered one of the factors that complicate pain management. Given that pain intensity ratings were positively correlated with the severity of nicotine withdrawal symptoms [15], controlling these symptoms is important not only in smoking cessation interventions but also in pain relief care. Smokers will benefit from cessation interventions that are tailored to meet their specific conditions.

#### Is smoking involved in opioid-related problems?

**< Summary Statements > **

- Opioid analgesic use is more prevalent in smokers than nonsmokers.
- Opioid analgesic consumption is higher in smokers than nonsmokers.
- Smoking is more common in patients with opioid use disorders than patients without.
- Young smokers have higher rates of opioid analgesic use and increased risk of opioid use disorder.
- Opioid analgesics do not reduce a risk of withdrawal symptoms after smoking cessation.

**< Commentary >**

Since both tobacco and opioid analgesics are dependent substances that activate the brain reward system, the use of opioid analgesics in current or former smokers is often a topic of scientific discussions. Previous reports indicated that smokers had more high risk for prevalence rates of opioid analgesic use [120–122], opioid analgesic use consumption [84, 111, 123, 124], and opioid use disorders [104, 122, 124, 125], compared with nonsmokers.

Therefore, it is important to explain and educate about smoking cessation to smokers, in order to minimize the amounts of opioid analgesics in patients requiring pain relief.

In addition, if prescribing opioid analgesics for pain relief is considered, a more careful approach, including the indication of opioid analgesic use, will be needed, in order to prevent opioid analgesic misuse and transition to opioid use disorder. Risk factors for opioid use disorder among primary care patients include smoking history at a young age, unemployment, multiple drug use [121, 122, 126], and cancer pain [104].

Since both smokers with and without opioid use disorder showed similar degrees of withdrawal symptoms of smoking cessation [127], it should be assumed that opioid analgesics do not reduce nicotine withdrawal symptoms.

### Impact of smoking cessation on pain sensation (significance of therapeutic intervention)

#### Does smoking cessation help improve pain?

**< Summary Statements >**

**Recommendation:** We strongly recommend smoking cessation interventions to help improve chronic pain. **[1C].**

- Smoking cessation helps alleviate pain by preventing exacerbation of underlying causes.
- Nicotine withdrawal symptoms arising from smoking deprivation may temporarily elevate pain sensitivity.
- Although smoking cessation can reduce pain and improve responsiveness to pain treatment in patients with certain types of disease, it is not clear whether smoking cessation provides positive short-term pain relief benefits in a wide spectrum of patients with chronic pain.

**< Commentary >**

Smokers experience more severe musculoskeletal and other chronic pain than nonsmokers because smoking causes tobacco-related diseases; damages musculoskeletal and other systems, leading for instance to osteoporosis, lumbar disc disease, and delayed bone and wound healing; and induces neuronal changes in pain pathways [11]. However, smoking cessation was not identified as an independent predictor of pain symptom changes in many studies [128, 129]. Chronic pain patients who smoke often fail to experience pain relief when they stop smoking. This is partly because they use tobacco as a means to cope with stress and depression, and hence nicotine withdrawal increases their pain perception. Tobacco smoking has acute analgesic effects that interfere with smoking abstinence efforts [10]. By contrast, studies in several clinical settings found that patients who quit smoking reported significantly greater improvements in pain and significantly better responses to treatment [32, 130]. Altogether, the available evidence does not allow us to definitively conclude that smoking cessation readily alleviates chronic pain. However, smoking cessation obviously can help suppress the aggravation and progression of tobacco-related diseases and other pain-causing pathologies, and its long-term benefits are undeniable. Therefore, it is the responsibility of pain practitioners to determine how they can help improve their patients’ pain-causing conditions and their healthy and overall life expectancy. To this end, they should share available evidence with their patients to decide on optimal pain and disease control strategies. Pain practitioners and smoking cessation therapists should develop patient awareness and education programs and treatment protocols to help patients achieve pain alleviation, complete freedom from smoking, and other goals.

The recommendation was based on the result of systematic review and meta-analysis [128]. The overall quality of evidence is low because of the imprecision and inconsistency of the results. The desirable effects of smoking cessation clearly outweigh the undesirable effects. Therefore, we strongly recommend smoking cessation interventions to help improve chronic pain. [1C].

#### Can freedom from smoking be achieved in patients with pain?

**< Summary Statements > **

- Patients with pain are often refractory to smoking cessation interventions due to high nicotine dependence and psychiatric/psychological complications.
- Patients with pain require intensive interventional programs that include pain relief care, education emphasizing that smoking does not reduce pain, and assistance in coping with anxiety.

**< Commentary >**

Nicotine provides acute analgesic effects [10]. Consequently, smoking patients tend to use tobacco to relieve or avoid pain [11, 131]. Repeated use of tobacco to avoid pain increases the risk of nicotine dependence. In addition, patients with pain are anxious about recurrence and exacerbation of pain. This anxiety triggers smoking behavior, further increasing the risk of nicotine dependence. Pain patients who smoke tend to experience depression and have difficulty and low confidence in their ability to quit or remain abstinent [132–134]. These findings indicate that pain patients who smoke are refractory to stop-smoking treatment.

In smoking patients with pain, abstinence interventions should start by addressing their pain because persistent pain is one of the main reasons for abstinence failure. Patients reported greater interest in receiving cessation interventions after initiating treatment for chronic pain [135], and pain ratings prior to smoking cessation were a useful predictor for the risk of early smoking relapse [136]. Pain control is a key to successful smoking cessation in smokers with pain.

Patients should learn that smoking does not reduce pain. The expectation that smoking would help cope with pain predicted lower odds of abstinence [137]. In a randomized controlled study of laboratory-induced pain, participants reported a reduced urge to smoke when they were challenged about their idea that smoking would decrease pain [138].

Pain-related anxiety was significantly associated with smoking dependence measures, and was an independent risk factor motivating smoking behavior [139, 140]. For these reasons, pain-related anxiety should be addressed in smoking cessation interventions for patients with pain.

Pain patients who smoke are likely to become highly nicotine dependent because they use tobacco as a means to cope with their pain and anxiety [132, 133]. Therefore, they should undergo interventions that are more intensive than standard smoking cessation programs. Multidisciplinary smoking cessation programs that are specifically designed to address patients’ psychiatric problems, mobility, and daily activities may be necessary [134]. Given the finding that craving for cigarettes was significantly associated with pain intensity ratings [15], pharmacologic treatments, including smoking cessation aids, are recommended.

Smokers with chronic pain have been reported to show higher prevalence rates of mobility and activity impairments [134, 141, 142]. For patients with severely hindered mobility, cessation interventions in the form of over-the-phone counseling, telemedicine, and mobile applications can be an effective option.

#### Do smoking cessation aids contribute to smoking cessation in patients with pain?

**< Summary Statements >**

**Recommendation:** We strongly recommend the use of smoking cessation aids to help pain patients quit and stay quit. **[1B].**

- In smokers with chronic pain, nicotine withdrawal increases pain intensity and reduces the likelihood of abstinence. Therefore, the use of smoking cessation aids is advised.

**< Commentary >**

Smokers with chronic pain use a larger number of cigarettes per day and are more likely to become nicotine dependent than those without chronic pain. If they have previously experienced difficulties when trying to stop smoking, severe nicotine withdrawal symptoms will probably develop on their next attempt [133]. Patients with chronic pain are resistant to smoking cessation treatment because pain intensities are positively correlated with desire to smoke [143]. Therefore, smoking cessation interventions should include effective pain control measures [144]. As smokers with chronic pain are amenable to using pharmacotherapy for smoking cessation [145], physicians should consider pharmacologic treatments to prevent nicotine withdrawal symptoms. Under the Japanese National Health Insurance System, smoking cessation programs cover cigarette smokers and HTP users.

First-line pharmacologic therapies for smoking cessation include nicotine replacement therapy (NRT), the partial nAChR agonist varenicline, and bupropion sustained release (an antidepressant that inhibits noradrenaline and dopamine reuptake). Various types of NRT (gum, transdermal patch, nasal spray, inhaler, sublingual tablets/lozenges) [146] and varenicline [147] can help improve the success rate of smoking cessation. In Japan, nicotine patches, nicotine gum, and varenicline have been approved for marketing.

It is unknown what types of smoking cessation aids are most effective in patients with chronic pain. One pilot study provided preliminary data suggesting the positive detoxification effects of varenicline in opioid-dependent adults with chronic pain, despite the absence of improvements in pain severity and depression [148]. In a study using murine pain models, varenicline demonstrated antinociceptive effects, indicating its potential as a treatment for chronic pain disorders [149]. A Cochrane review was conducted regarding antidepressants’ smoking cessation effects, and showed that bupropion sustained release and nortriptyline significantly increased success rates [150]. A placebo-controlled crossover study reported that bupropion sustained release (150–300 mg/day) effectively improved neuropathic pain [151], but this product has not currently been approved for marketing in Japan. Nortriptyline is often prescribed in Japan for treating pain. Its pain-relieving effects may highlight its utility as a smoking cessation aid. Considering that serotonin noradrenaline reuptake inhibitors may have fewer side effects than other types of antidepressants, they may be useful as smoking cessation aids in patients with pain, particularly those with diabetic peripheral nerve disorders and lumbago. Future research is warranted.

Other important components of smoking cessation interventions for smokers with chronic pain include stress mitigation during pain treatment and careful management of prescription opioids [152]. Moreover, given the importance of pain physicians advising and assisting patients to quit smoking in their practices, an educational module about smoking and pain should be incorporated in pain medicine training programs [153].

The recommendation was based on the previous Cochrane systematic reviews [146, 147]. The overall quality of evidence is moderate because of indirectness of evidence (no study in patients with chronic pain context). The desirable effects of smoking cessation aids clearly outweigh the undesirable effects Therefore, we strongly recommend the use of smoking cessation aids to help pain patients quit and stay quit. [1B].

#### Does cognitive behavioral therapy (patient education) assist smokers with pain?

**< Summary Statements > **

- Multidisciplinary pain management programs that include cognitive behavioral therapy (CBT) can be applied to smoking cessation treatment.
- Behavioral therapy including smoking cessation in combination with physical therapy can be a first-line intervention for patients with chronic pain.

**< Commentary >**

Many patients with chronic pain have difficulty quitting smoking. However, CBT can help increase the odds of quitting, allowing them to complete multidisciplinary pain treatment at a higher success rate. Therefore, integrating CBT-based smoking cessation interventions into pain therapy can be an effective treatment program for smokers with chronic pain [154].

On the other hand, a systematic review of smoking cessation interventions for patients with chronic pain, including pharmacologic treatments, physical activity programs, and behavioral and psychological therapies, suggested that they could possibly change the smoking status and reduce the number of cigarettes consumed per day, but albeit with no effects on pain, physical function, depression, or anxiety [128]. Moreover, in a randomized controlled study that compared standard and individually tailored intensive smoking cessation programs in patients with rheumatoid arthritis, both programs reduced the mean number of cigarettes smoked per day (despite the absence of significant differences between programs in quit rate, pain intensity, physical functioning, mood, and QOL) [155].

These findings suggest the utility of CBT-based multidisciplinary pain treatment programs in assisting with smoking reduction. However, the patients with chronic pain often rely on smoking as a means to cope with pain and distress, and a large proportion of patients who use opioid analgesics for pain relief report that opioid consumption stimulates smoking. Important barriers to making a quit attempt during pain treatment included making changes in opioid dose while quitting and perceived difficulty managing multiple treatment-related stressors [152].

Under these circumstances, behavioral therapy for behavioral change including smoking cessation either independently or in combination with physical therapy, can be a first-line intervention for patients with chronic pain [156] (for more details, refer to the next section). CBT can be effective for pain management and smoking cessation. However, its effectiveness for smoking cessation in patients with pain and managing pain in smoking patients must be confirmed in future studies.

#### Does exercise therapy (physical exercise and activity programs) assist smokers with pain?

**< Summary Statements > **

- Exercise-induced hypoalgesia (EIH) is mediated by the brain reward system in a manner similar to nicotine-induced acute analgesia. However, EIH is not addictive, and provides promising long-term effects.
- Smoking cessation and physical exercise both improve disease activity, physical function, and QOL, whereas smoking interferes with pain rehabilitation effects.
- Physical inactivity, smoking, and other unhealthy lifestyle factors increase the risks of chronic pain.
- Lifestyle management programs that encourage smoking abstinence, physical exercise, and other behavioral changes, administered either alone or in combination with conventional physical therapy, can be a first-line intervention for patients with chronic pain.

**< Commentary >**

Nicotine has acute analgesic potential [11]. It activates both nAChRs and opioid receptors, which trigger the release of dopamine in the nucleus accumbens, the brain’s reward center (nicotine reward system). This process is similar to the mechanism of EIH. However, EIH evidently provides much more effective and healthier effects on pain control than smoking. Long-term adherence to exercise regimens can help improve pain control, physical function, and QOL in patients with chronic pain. A major difference between EIH and nicotine-induced analgesia is that EIH results in mostly endogenous pain modulation whereas nicotine absorbed from smoking serves as an exogenous agonist. This and other differences make physical exercise less addictive. In a study of smoking cessation in patients with ankylosing spondylitis, smoking cessation interventions significantly lowered disease activity and pain intensity, and increased physical functioning and QOL [130]. These findings indicate that smoking cessation can yield favorable health benefits similar to those of physical exercise. Moreover, in a study of patients with chronic spinal disability undergoing an intensive functional chronic pain management rehabilitation program, the percentages of patients who completed the program and those who retained work at 1 year post-treatment decreased with smoking levels, although no significant differences were noted in other socioeconomic outcomes at 1 year post-treatment [157]. Smokers reported slightly higher post-treatment pain and disability ratings. These results provided evidence that smoking negatively affects pain rehabilitation effects.

Pain is strongly associated with physical inactivity, smoking, and other unhealthy lifestyle patterns. Heavy-smoking individuals who were physically inactive tended to have greater odds of smoking when experiencing pain [26]. Individuals with chronic low back pain tended to have lower physical activity during leisure time and a higher body mass index [158]. Smoking, lack of exercise, physical inactivity, and other negative lifestyle patterns were risk factors for lumbago [159]. In addition, persistent chronic widespread pain was weakly associated with former smoking and overweight, although not with moderate exercise or alcohol use [28]. These results are consistent with those of another study reporting that smoking and other negative lifestyle factors were risk factors for chronic widespread (musculoskeletal) pain [160]. Given the inverse relationship between chronic pain (e.g., low back pain) and positive lifestyle behaviors such as smoking cessation and physical activity, behavioral change (lifestyle management) interventions that include smoking cessation support, administered alone or in combination with physical therapy, can be a first-line option for patients with chronic pain. They will also reduce the socioeconomic burden of chronic pain [156].

## Appendix

Search Formulae (July 2020)

MEDLINE (PubMed)

Keywords: “smoking,” “tobacco,” “smoking prevention,” “smoking cessation,” “smoking cessation aid,” “nicotine,” “tobacco use disorder,” “analgesia,” “analgesic,” and “pain.”

### Literature search was conducted in July 2020 using the following formulae:

**Table:**

#	Search Formula	Results
1	“Smoking Cessation” [MeSH] OR “Smoking Reduction” [MeSH] OR “Tobacco Use Cessation” [MeSH]	29,458
2	“Smoking Prevention” [MeSH]	17,979
3	“Smoking” [MeSH] OR “Tobacco Use” [MeSH]	147,867
4	“Tobacco Use Cessation Devices” [MeSH]	1800
5	“Smoking Cessation Agents” [MeSH]	148
6	Nicotine [tiab]	41,047
7	“Tobacco Use Disorder” [MeSH]	11,169
8	or/#1–#7	194,066
9	analges* [tiab]	129,715
10	analgesia [MeSH]	43,859
11	analgesics [MeSH]	190,769
12	“Acute Pain” [MeSH] OR “Chronic Pain” [MeSH] OR “Cancer Pain” [MeSH]	17,208
13	or/#9–#12	298,420
14	8 and 13	2003
15	Filters: from 2000 to 2020	1446
